# Polygenic risk and predominant polarity in individuals with bipolar disorder

**DOI:** 10.1192/j.eurpsy.2021.241

**Published:** 2021-08-13

**Authors:** S.H. Jensen, S. Østergaard, K. Musliner

**Affiliations:** 1 Department Of Affective Disorders, Aarhus University Hospital - Psychiatry, Aarhus C, Denmark; 2 Department Of Economics And Business Economics, Aarhus School of Business and Social Sciences, Aarhus C, Denmark; 3 Department Of Affective Disorders, Aarhus University Hospital - Psychiatry, Aarhus, Denmark

**Keywords:** bipolar disorder, predominant polarity, Polygenic risk

## Abstract

**Introduction:**

Individuals with bipolar disorder often have a ‘predominant polarity’ (e.g. depressive or manic) that characterizes the majority of episodes over the course of the illness. Genome-wide association studies have suggested a relationship between genetic risk and phenotypic heterogeneity in bipolar disorder. However, to date, no study has directly examined the association between polygenic liabilities and predominant polarity in bipolar disorder.

**Objectives:**

To estimate the associations between the polygenic risk score for major depressive disorder (PRS-MD), bipolar disorder (PRS-BD) and schizophrenia (PRS-SZ), and predominant polarity among individuals with bipolar disorder in hospital-based settings in Denmark.

**Methods:**

The study sample will include all individuals from the Initiative for Integrated Psychiatric Research (iPSYCH2015) sample who received a diagnosis of bipolar disorder and were successfully genotyped (approximately 3,400). Information on polarity will be computed based on data from the Danish Central Psychiatric Research Register. PRS variables will be generated using the most recent results from the Psychiatric Genomics Consortium. Odds ratios for the associations between PRS variables and polarity will be estimated using logistic regression.

**Results:**

We hypothesize that PRS-MD will be highest among the predominantly depressed patients, that PRS-BD will be highest among those with predominantly manic/mixed episodes, and that PRS-SZ will be highest among those who experience psychotic mania or psychotic bipolar depression. The results will be shown at the conference.

**Conclusions:**

A finding of association between genetic liability and predominant polarity in bipolar disorder could pave the way for stratification on genetic liability in future treatment studies and in clinical practice.

**Disclosure:**

No significant relationships.

